# Exploring facilitators and barriers to long‐term behavior change following health–wellness coaching for stroke prevention: A qualitative study conducted in Auckland, New Zealand

**DOI:** 10.1002/brb3.2671

**Published:** 2022-12-12

**Authors:** Caroline Holder, Rita Krishnamurthi, Alice Theadom

**Affiliations:** ^1^ National Institute of Stroke and Applied Neurosciences (NISAN) Auckland New Zealand; ^2^ School of Public Health & Psychosocial Studies Auckland New Zealand; ^3^ Faculty of Health and Environmental Sciences Auckland New Zealand

**Keywords:** health behavior change, health wellness coaching, stroke prevention

## Abstract

**Introduction:**

Health–wellness coaching (HWC) has grown in popularity as a means of empowering individuals to take responsibility for their health behavior and make lifestyle changes to reduce their risk of stroke. Understanding the facilitators and barriers to long‐term behavior change is key if preventive strategies such as HWC are to be robust and effective. This study aimed to explore the experiences of people at risk of stroke after receiving HWC for stroke prevention, specifically the facilitators and barriers to long‐term behavior change from the perspective of study participants.

**Methods:**

All participants received HWC as part of a randomized controlled trial 3 years earlier. Semi‐structured telephone interviews were conducted with eight participants from the trial sample. Interviews were audio‐recorded and transcribed verbatim. Reflexive thematic analysis was used to identify key concepts and themes.

**Results:**

Three overarching themes were identified: “Awakening of the mind” captured the importance of seeing the bigger picture, recognizing the impact of potential disease and using skills and tools to support decision‐making. “It's not just about health behavior” conveyed the importance of being respectfully responsive to individual need and addressing emotional well‐being alongside physical health. “Social connectedness” encapsulated the significance of community engagement, accountability, and paying it forward.

**Conclusions:**

Enhancing awareness of personal risk and the impact of potential disease are facilitators of long‐term behavior change and should be incorporated into coaching conversations. This supports the process of “waking up” to health needs and the possibility of change, which are important precursors to long‐term change. Health coaching should be responsive to individual need, with emotional well‐being, happiness, and life satisfaction being addressed alongside physical health. The opportunity to develop skills to support decision‐making and self‐management should be included in coaching initiatives, to enhance self‐efficacy and help facilitate long‐term behavior change.

## Graphical abstract

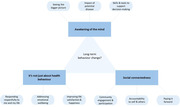

## 1 INTRODUCTION

Stroke is the second leading cause of death worldwide and the third leading cause of death in New Zealand (Feigin et al., 2017). It can result in physical disability, impairment in social and cognitive functioning, mood changes and fatigue, impacting daily living activities on a long‐term or permanent basis (Barker‐Collo et al., 2016; Kapoor et al., 2017; Persson et al., 2017). Accordingly, there has been renewed emphasis on reducing stroke burden via effective stroke prevention. Effective lifestyle and behavior modification could prevent around half of all strokes (Tikk et al., 2014). The INTERSTROKE study demonstrated that stroke is largely preventable, and that 90% of strokes could be accounted for by 10 modifiable risk factors, including smoking, lack of physical activity, poor diet, and drinking alcohol (O'Donnell et al., 2016). Furthermore, evidence has shown that the presence of ideal cardiovascular health factors is associated with a lower risk of stroke (Kulshreshtha et al., 2013). Management of these cardiovascular risk factors by adopting a healthy lifestyle is prudent in stroke prevention. Despite growing a knowledge of the barriers and facilitators that are likely to predict uptake of lifestyle behavior change (Alageel et al., 2018; Graham et al., 2014; Neuner‐Jehle et al., 2013), relapse rates for those achieving change remain high (Furguson et al., 2005; Curioni & Lourenco, 2005). Facilitators and barriers that influence the long‐term *maintenance* of behavior change are even less well understood. A systematic review of 22 qualitative studies by Murray et al. (2013) explored the barriers and facilitators of maintaining healthy lifestyle behaviors. They reported that social support, education, knowledge, beliefs, and emotions are key influences in this and are important for maintaining healthy behaviors in the longer term (Murray et al., 2013). However, this study was not specific to behavior change after Health–wellness coaching (HWC); therefore, the facilitators and barriers reported cannot be assumed to be relevant to behavior change after HWC.

HWC is a client‐centered, goal‐oriented partnership in which a coach helps a person to identify personal strengths and motivates them to focus on lifestyle behaviors they want to change (Lindner et al., 2003). Research into the effectiveness and feasibility of coaching within health‐care settings has shown promise (Neuner‐Jehle et al., 2013), but results have been inconsistent (Askim et al., 2008). HWC may well have applicability to improve lifestyle behavior and assist in primary stroke and cardiovascular disease prevention; however, there is a lack of qualitative research into which influences may help and hinder long‐term behavior change once the coaching has finished (Gallacher & Quinn, 2018). The current study aimed to fill this gap by enhancing understanding of the facilitators and barriers to long‐term behavior change following real‐life experiences of HWC. Reflexive thematic analysis (Braun & Clark, 2019) was used with the theoretical motive to maximize the integrity of the research process, while allowing the researcher to be subjective. In this research study, reflexive thematic analysis is suited to studying the lived experience of people who had received health coaching, and the factors that influenced their health behaviors. The results of this study may be used to further develop coaching initiatives for stroke prevention and therefore has implications for clinical practice.

## 2 AIMS

This study aimed to explore the facilitators and barriers that may facilitate or hinder long‐term behavior change in people after HWC for stroke prevention.

## 3 METHODS

Ethical approval was obtained from the Health and Disability Ethics Committee (16/NTA/36) and Auckland University of Technology Ethics Committee (AM03/AUTEC).

### 3.1 Recruitment

Participants were recruited from a larger study previously conducted within our team, which we will refer to from here on in as the parent study. The parent study was undertaken to evaluate the effectiveness of HWC in populations at an increased risk of stroke PREVENTS (Mahon et al., 2018). The parent study included 320 adults with absolute 5‐year cardiovascular disease risk ≥10%, calculated using the PREDICT web‐based clinical tool (Riddell et al., 2010). Half of the participants were randomized into a control group and the other half into an intervention group receiving up to 15 sessions of HWC over a 9‐month period. Study participants were followed up at 3‐, 6–9‐, 12‐, and 36‐month post‐randomization. During the PREVENTS study, five coaches with various professional backgrounds were trained in HWC over several weeks, and each coach took part in a monthly group supervision session with an accredited coaching professional, in order to discuss coaching practice and ensure intervention integrity. The coaching intervention included the use of a resource pack that included a self‐assessment for several areas of well‐being, such as physical, emotional, social, occupational, environmental, and spiritual well‐being. Coaching participants would score themselves out of 10 in each area, state what scores they would like to achieve by the end of the coaching, and discuss what changes may need to take place in order to achieve those new scores. These, along with a values‐based activity, were required to be used at the beginning of each and every coaching intervention as they provided a road map for the coaching relationship, ensuring that the intervention plan was focused on meaningful goals that were relevant to each individual. Other tools within the resource pack included goal‐setting and problem‐solving templates, weekly planners, resources to assist with time‐management and organizational skills, emotional regulation tools, and a diary. The same resource pack was given to each participant during session one, but aside from the two compulsory activities that were completed at the beginning of each coaching intervention, the resources and tools used varied for each participant depending on their individual needs and goals.

A subset of nine participants from the PREVENTS study were approached and invited to take part in this study, having been selected for convenience based on their chronological randomization into the coaching intervention group. They were initially approached via telephone and, if agreeable, were then sent a participant information sheet and consent form via post. Of the nine approached, eight gave informed consent, and one declined to take part due to not being interested. Participants were eligible to take part in this study regardless of how many coaching sessions they had completed. Table 1 below includes a column that details the number of coaching sessions each participant completed, thereby giving an indication of the varying adherence levels between the eight study participants. The sample size was determined to capture diversity in age, sex, number of sessions completed, and CVD risk (see Table 1). On the review of the eight cases selected, sufficient breadth of experience on these characteristics was deemed to have been achieved, and no further recruitment was needed. Although attempts were made to recruit as many participants from diverse ethnic groups as possible, the Chinese Asian population had greater rate of refusal in the original sample. Consequently, the Chinese Asian population are underrepresented in both the parent study and in this study.

**TABLE 1:** Participant characteristics

Participant	Age	Sex	Ethnicity	Sessions completed	CVD risk (%)
1	50–59	M	Cook Islands Maori	5	26 (High)
2	60–69	M	Indian	10	13 (Mod)
3	40–49	F	Indian	7	15 (Mod)
4	50–59	F	Cook Islands Maori	10	12 (Mod)
5	60–69	M	NZ Euro/Maori	4	18 (High)
6	60–69	F	Cook Islands Maori	11	11 (Mod)
7	70–79	M	NZ Euro	15	30 (High)
8	70–79	F	Maori	15	11 (Mod)

### 3.2 The interview process

Semi‐structured one‐to‐one telephone interviews, conducted by an experienced interviewer, were undertaken with the eight participants. The interviewer was employed as a clinical researcher on the team and had previously been one of the coaches for the PREVENTS study. However, none of the participants had been coached by the interviewer, and none were previously known to her in any other capacity. In order to reduce the risk of the interviewer's previous role influencing participant answers, the interviewer's previous involvement in the PREVENTS study was not disclosed to study participants. The interviewer had extensive experience conducting interviews for research purposes, including one‐to‐one interviews (structured and semi‐structured), questionnaires, and conducting focus groups. An interview guide was used for the interviews, which was produced by senior members of the research team. The sample questions have been submitted as supplementary information to this article. Interviews were audio‐recorded and transcribed verbatim. Interview notes were taken by the interviewer to capture key observations to support analysis. These observations were discussed extensively within the research team in order to ensure integrity and avoid confirmation bias.

Some participants completed their telephone interviews at home, whereas others completed them at their place of employment during lunch breaks or after work. All were completed telephonically with no other persons present other than the interviewer and interviewee. The mean interview duration was 12 min, the shortest being 9 min and 16 s and the longest being 18 min and 47 s, respectively. Participants were given the opportunity to review the recordings upon request for the purpose of clarifying or amending their responses. None of the participants requested to do so. The aim of thematic analysis is to ensure that the sample size is small enough to enable an in‐depth understanding of the phenomenon, whilst large enough to capture breadth of experience (Sandelowski, 1995). Upon the completion of the eight interviews, consideration was given as to whether more participants were required, but following in‐depth discussions between the authors, it was agreed that saturation point had been reached, and that the sample size was large enough to adequately answer the research question.

### 3.3 Qualitative approach

Reflexive thematic analysis (Braun & Clarke, 2006) was used to analyze and interpret the data. No themes were discussed in advance of the interviews, all of them derived organically from the data. To ensure rigor and reflexivity, two researchers independently reviewed the transcribed interviews, first familiarizing themselves with the data, then noting key ideas and creating initial codes. These codes were collaboratively grouped together in clusters of meaning. Care was taken to ensure the initial codes accurately represented the transcripts from which they were taken, with changes being made where needed to ensure code names accurately reflected the diversity of experience within the raw data. To further ensure rigor, the revised codes were discussed with a third researcher who made further checks to ensure integrity, accuracy, and consensus. All three researchers collaboratively arranged the final codes into themes and subthemes that were mapped out to explore relationships between them. Final themes were discussed and agreed as the truest representation of the data findings.

## 4 RESULTS

Participants described many barriers and facilitators to long‐term behavior change. Some factors were reported as being directly related to HWC, whereas others were described as a result of more general life influences, or a combination of both. Accordingly, all changes made and maintained during the 3‐year follow‐up period cannot be attributed directly to the HWC.

### 4.1 Themes

**FIGURE 1:**
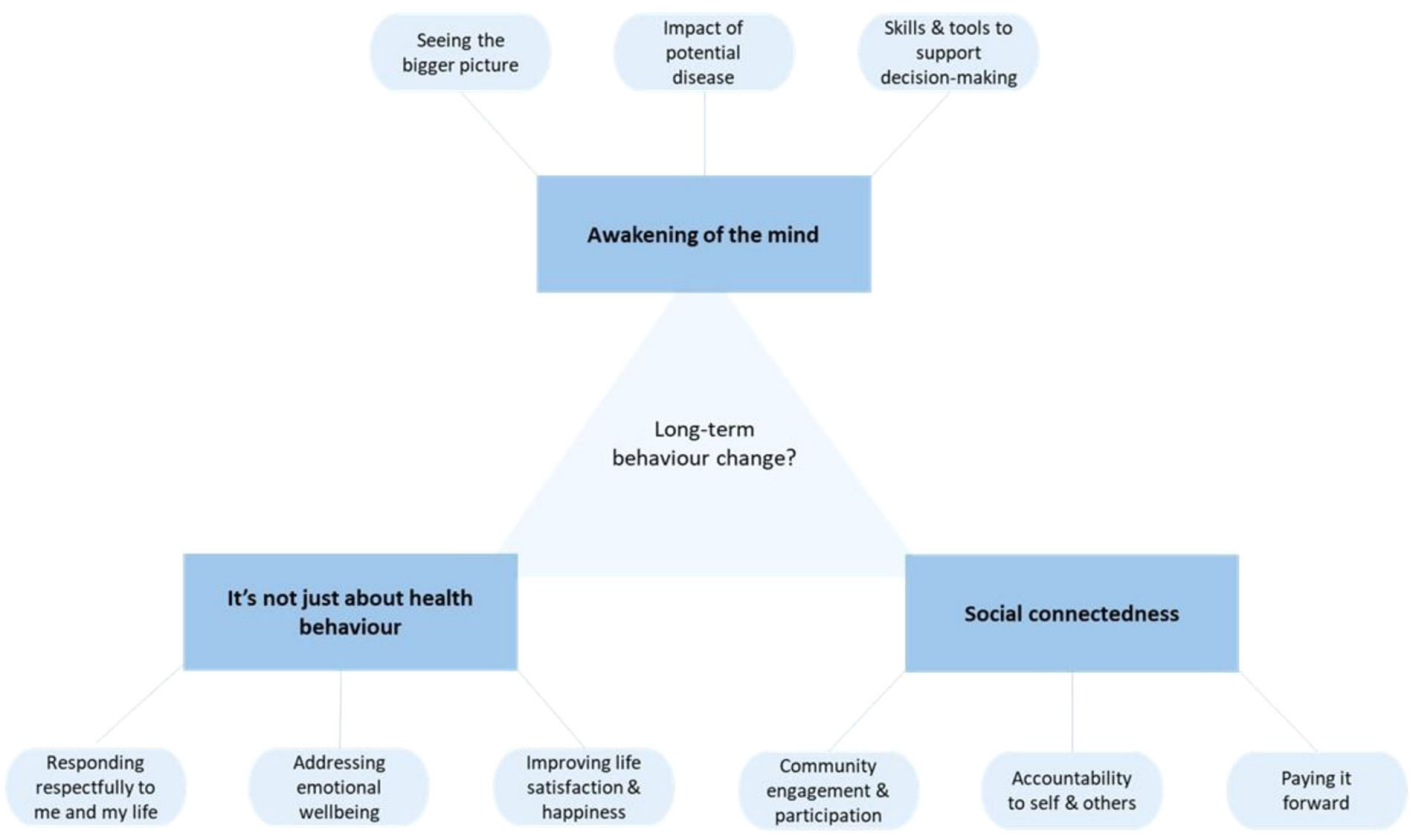
Themes and subthemes

Three overarching themes and nine subthemes were identified as being relevant to the achievement or otherwise of long‐term behavior change (Figure 1). The results are supported by direct participant quotes with participant reference number in brackets (linked to Table 1).

#### 4.1.1 Theme 1—“Awakening of the mind”

##### Seeing the bigger picture

A number of participants talked about “waking up” to their health risks, and this being a major driver for change and long‐term maintenance. Some attributed the process of “waking up” directly to the coaching, saying it brought greater awareness and an ability to see the bigger picture:

“…it's just like [an] awakening of the mind…Everything changes.” (P3, Female, Moderate CVD risk)

This participant described how prior to the coaching she only focused on the needs of other people, often to the detriment of her own health needs. She talked about a profound realization that in order to take care of her family, she needed to take care of herself first:

“…before the coaching I just used to think about my children's needs, my family, my husband…this thing just helped to, ok, if I be ok, if I'll be here, if I have got a long life…then I can do things for my kids.” (P3, Female, Moderate CVD risk)

In learning to prioritize herself, this participant reported a healthier balance between family commitments, study, and her own health goals, which she was able to maintain in the long term.

Some described waking up to their health risks more naturally following diagnosis, with HWC contributing little toward this, whereas others described the timing of HWC as highly significant, being either optimal or not. For instance, P5 attributed his early withdrawal from the coaching to becoming “quite busy” and therefore too “tired” to engage:

“…all I wanted to do was come home and sleep” (P5, Male, High CVD risk)

Conversely, P3 described the timing of her coaching as ideal as it came at a time in her life when she really needed it and was ready to make change:

“…and that's the time I started this coaching…which was quite, quite good…that's the time I really needed somebody to you know, help me, help me out…” (P3, Female, Moderate CVD risk)

The timing of HWC then may influence the effectiveness of the intervention in terms of a person's ability and readiness to make change as well as their ability and motivation to maintain it.

##### Impact of potential disease

Perception of personal risk relating to current health conditions and awareness of the impact of potential disease varied among participants. For some, insight into the possible future impact of current health conditions on quality of life and independence was cited as a motivating factor for maintaining positive behavior change:

“…I'm much more aware of the problems that I could face…organs could be failing, liver, kidney, things like that…so I continuously try and look out for things that's going to be good for me, especially diet wise.” (P2, Male, Moderate CVD risk)

Fear of losing physical functioning, independence, and becoming a burden on others was discussed by many participants as reasons for being proactive and maintaining health behavior change:

“…once you lose your health…then you are incapable of doing things and…become a burden for other people…for my family…for the government…I realise that it's me, that I can look after myself…I have to live a healthy life, I don't want to be bedridden” (P2, as above)

Although the awareness of personal risk was a motivating factor for some, for others, a lack of insight into the potential impact of disease was apparent:

“…I feel that there's nothing really wrong with me, I'm strong and believing that I'm not sick, I think it's all these medications that are making me sick” (P6, Female, Moderate CVD risk)

For this participant, poor insight into personal risk and the potential impact of disease was cited as a reason for poor medication adherence, and for others, it was cited as a reason for early withdrawal from the coaching.

##### Tools and skills to support decision‐making

Using tools and developing new skills to support decision‐making was cited as a facilitator of long‐term behavior change by many participants. These included skills in prioritizing self, time‐management, goal‐setting, problem‐solving, establishing routines, and coping skills:

“…I was learning how to cope…well I was coached into looking at the bigger picture, trying to prioritise what was important to me…make some good decisions…” (P4, Female, Moderate CVD risk)

Using time management skills to schedule in exercise proved difficult for some to maintain over time, with life and busyness often getting in the way:

“So I sort of find that hard to put in with the erm, doing kura [study] and then the other things I get involved in” (P8, Female, Moderate CVD risk)

Something that helped maintain long‐term change for some participants was the ongoing use of the coaching tools and resources described in Section 3. Many kept these resources to hand after the coaching had ended, revisiting them occasionally as a reminder of the skills learnt or as a refresher when facing particular challenges:

“…having the book, and record of it as I was having the coaching sessions has helped…there are times when I thought well what would I do here, and I'd pick up the book and read it and see…look at the book itself and what she would tell me to do, and I thought yeah I'll try that” (P8, as above)

A number of participants made similar comments about the usefulness of keeping a record during the coaching and keeping the resources and tools to refer back to.

#### 4.1.2 Theme 2—it is not just about health behavior

##### Responding respectfully to me and my life

In discussing what made the HWC meaningful, many participants discussed the whole person approach and valued that the coaching was not just focused on physical health:

“…I've seen in that coaching, it's not just the health…It's the health and wellbeing…your lifestyle, your motivation, everything comes in this coaching, that is the good part…if you just come and you talk about your health, health, health, people think…why…” (P3, Female, Moderate CVD risk)

Some participants took action to improve work–life balance, occupational satisfaction, and boundaries within relationships, changes that were often seen as important in improving happiness and maintaining positive health behaviors. Participants also valued having a trusting and respectful relationship with their coach:

“…the coach becomes more like family…someone you can trust” (P8, Female, moderate CVD risk)

When asked about cultural safety and whether consideration should be given to matching coaches and clients based on ethnicity or cultural background, conflicting views were expressed. Most participants felt it would be a good idea to offer this option, as for some, it would be very important, particularly in New Zealand where there is rich cultural diversity:

“I think definitely there would be some people that would prefer it…especially in some ethnic groups, they may feel intimidated and they'll say yes to anything…it may be because sometimes they don't understand the question…culturally” (P1, Male, High CVD risk)

However, some raised concerns about confidentiality in small communities and suggested that best practice would be to consult with coaching participants individually on this rather than making assumptions, as a good connection may not be dependent on shared characteristics.

##### Addressing emotional well‐being

Many participants valued “having someone to talk to” as a benefit of coaching. Some described having no‐one else to talk to about personal issues, whereas others described a coach as being different to talking to a doctor or health professional:

“…I find what is very useful is somebody to talk to…sharing about how I feel, to do with my health, to do with the coaching, where I can improve, what can I do next, that kind of thing…I find that with the doctors…it's not the same…” (P6, Female, Moderate CVD risk)

Some participants needed to deal with issues effecting their emotional well‐being before being able to take steps toward improving their physical health and valued that the coaching facilitated this:

“…when she first came here…I was quite down…But during this coaching I mean, I'm more positive…I would think I will do this, I can do this…I can get my diabetes under control…” (P3, Female, Moderate CVD risk)

For this participant, improved emotional well‐being, positivity, and a greater sense of self‐efficacy were all cited as facilitators of long‐term behavior change.

##### Improving life satisfaction and happiness

For some participants, significant life events had a more profound impact on long‐term change than the coaching itself. P1 described experiencing multiple early deaths within his community, which evoked a sense of wanting to do something more fulfilling and meaningful with the remainder of his life. This instigated a change in his employment to achieve more alignment with his values and therefore a greater sense of purpose, life satisfaction, and happiness:

“I don't believe that money brings true happiness or health in life…the satisfaction I get out of…helping the community when we do projects, the outcome of just seeing the faces of the people smiling…just that communal type of thing you know…” (P1, Male, High CVD risk)

This participant discussed being happy with his general health as the coaching ended. However, he was most proud of the major life change he made to his employment, which was a big part of his overall well‐being. Participants also described how experiencing and recognizing the benefits of change may also make long‐term change more likely:

“I don't wanna go back…because I notice the difference in my body with all that coaching…the improvements…it makes me feel a lot better and healthier…” (P6, Female, Moderate CVD risk)

This participant lost 10 kg during the coaching and another 2 kg post‐intervention. She cited feeling happier and healthier as motivating factors in helping her to maintain the dietary changes made.

#### 4.1.3 Theme 3—social connectedness

##### Community engagement and participation

Community engagement and participation was regarded as a facilitator of long‐term health behavior change for many participants. For instance, P6 described the benefits of being busy with voluntary work and paid employment:

“It keeps me really busy and not think about being at home and eating and get fat” (P6, as above)

Conversely, these activities created barriers in other areas of well‐being for this participant, for example, she had little time or energy to maintain exercise routines. However, she worked around this in the long term by incorporating walking into her working day:

“…as long I am doing my walking, as long I am eating healthy…as long as I'm drinking plenty of water…I am doing all of those things and I am happy” (P6, as above)

Increased community engagement appeared to bring greater happiness and life satisfaction for many participants. However, for others, it was more important to be around like‐minded people that have similar health goals, particularly when trying to get back on track after a lapse:

“…I need to be around people who are involved in it…that would be the chatter around me so it would probably get me going…” (P8, Female, Moderate CVD risk)

This highlights the potential influence of friends and social networks in long‐term behavior change, suggesting that the attitudes and interests of those we engage with socially may support or sabotage the maintenance of healthy habits.

##### Accountability to self and others

Many participants described accountability as a major facilitator of change during the coaching process, stating that having someone call them to discuss their progress toward goals felt like a form of checking‐up, making them more likely to complete agreed upon actions:

“I'd get a call or something and then I'd think ok, ok, I've got to be good, I've got to be good, they're checking up on me…” (P4, Female, Moderate CVD risk)

Those who found ongoing ways to stay accountable for their health goals after the coaching ended had more success in maintaining the changes made. This contrasts with participants taking less personal responsibility for their health, which was synonymous with less self‐control and more likelihood of relapse:

“…what tends to happen is it's you and your partner in the truck and you go past and if she's hungry you've got to stop at a bakery…and then I'm back into it again” (P5, Male, High CVD risk)

P2 repeatedly emphasized that people are responsible for their own health but need help to be able to self‐manage, indicating the need for both ongoing support and personal accountability in long‐term health behavior change.

##### Paying it forward

Many participants described “paying it forward” by taking on “coach‐like” roles with others both during the coaching and as it had ended. P7 had set up an exercise program, P3 talked about coaching fellow students and family members, and P8 and P6 discussed coaching extended family on stress management and healthy food choices:

“…sometimes when I see them drinking…sweet drinks, even my own grandchildren, I'm encouraging them, please just give them water” (P6, Female, Moderate CVD risk)

It is of interest that within this study, those who were “paying it forward” in some way were also maintaining some form of health behavior change, a concept that is discussed further below.

## 5 DISCUSSION

“Waking up” to the need for health behavior change appears to be a crucial precursor to and facilitator of long‐term health behavior change. Seeing the bigger picture, recognizing health risks and understanding how those risks might affect disability, level of independence, and quality of life were all acknowledged as important. This supports previous findings on the association between the patient awareness of their risk factors and their ability and readiness to change behaviors over time and reduce their risk of subsequent stroke (Eames et al., 2011). Conversely, a lack of insight into these areas was associated with disengagement from coaching and/or lapse and relapse of some or all health‐related goals, thus presenting barriers to long‐term change. Discussions that deepen understanding of personal risk and the impact of potential disease should therefore undoubtedly be incorporated into HWC conversations to help facilitate long‐term change. The concept of “waking‐up” was also associated with the development of a positive attitude by some participants, which included a greater sense of self‐efficacy, shown previously as an essential component of change (Holloway et al., 2002). Enhancing self‐efficacy then is perhaps intrinsic to HWC, an idea that aligns with the findings of this study and is supported further by Lennon et al. (2018). In their review of interventions for behavior change and self‐management in secondary stroke prevention, Lennon et al. (2018) put forward a comprehensive model for effective person‐centered interventions, in which risk factor knowledge, mind–body awareness, and individualized behavior change plans are highlighted as essential components of self‐efficacy, self‐management, and long‐term behavior change.

Using tools to support decision‐making and learning new skills in prioritizing self, establishing routines, goal‐setting, problem‐solving, and time‐management were found to be significant facilitators of long‐term behavior change. Similar findings were reported in an employer‐offered pilot study of HWC coaching where goal‐setting, improved personal awareness, and motivation were seen as key values of health coaching (Nelson et al., 2020). Some of the skills and tools discussed by participants were acquired via HWC, whereas others were learned more organically through life experiences. Nevertheless, an opportunity to upskill and use tools to support decision‐making could enhance HWC programs. This finding is again supported by the model put forward by Lennon et al. (2018), which hails the importance of acquiring knowledge and skills in order to self‐manage in the long term and achieve positive primary and secondary health outcomes. The ongoing use of coaching resources was significant for many participants for the purpose of retaining skills for long‐term self‐management. This reinforces the previous finding that providing self‐management support is a key component of HWC (Bennett et al., 2010). Keeping any coaching resources or tools used during the coaching intervention should therefore be encouraged so that participants have something tangible to refer back to as a refresher or a reminder of the tools used and self‐management skills learned. A further finding of this study is that the timing of HWC should perhaps include the consideration of any competing demands on the participant and also their readiness to commit to long‐term change, thus allowing resources to be allocated to those most likely to benefit.

A key finding is that HWC should not just focus on physical health; it should be person‐centered, responsive to individual need, and inclusive of all areas of well‐being, further illustrating the model put forward by Lennon et al. (2018) discussed above. In particular, improving emotional well‐being and addressing problems affecting life satisfaction and happiness were valued by study participants and seen as important facilitators of long‐term change. This aligns with a quantitative study of health behavior changes after HWC, which found that participants observed improvements in their overall quality of life, spiritual well‐being, and reduced negative emotions among others following HWC (Clark et al., 2016). It is also supported by the recommendation that providing emotional support is one of the five fundamental roles of a health coach (Bennett et al., 2010). HWC initiatives may therefore be significantly enhanced by focusing on both physical and emotional well‐beings to help facilitate long‐term maintenance of behavior change. It has also been suggested that, incorporating support for other barriers that could impact on quality of life, such as improving sleep and managing stress, may enhance HWC further by contributing additional support not usually provided in a clinical setting (Perlman & Dabrh, 2020). A trusting and positive relationship with the coach was important to participants and may have implications not only for level of engagement, but also for the achievement or otherwise of long‐term health goals. Although the quality of the relationship was not dependent on shared ethnicity, participants strongly felt that they should be consulted individually about whether they would like this to be the case. This would help to ensure cultural safety whilst also helping to create a positive and collaborative relationship based on trust and mutual respect.

Community engagement was seen as a good distraction from unhealthy habits, whilst also potentially being a barrier in terms of participants being too busy with community participation to follow through with planned exercise routines. Overwhelmingly though, community engagement and participation appeared to have a positive impact on self‐esteem, identity, fulfillment, and happiness, which in turn impacted on long‐term motivation to stay well. This is not surprising as community participation and social connectedness have been shown to be associated with enhanced mental well‐being (Ding et al., 2015). Social supports and being around like‐minded people with shared goals were repeatedly cited as important facilitators of long‐term behavior change. These facilitators should therefore be encouraged within future coaching programs to increase the likelihood of long‐term behavior change being achieved. Where appropriate, social connectedness and support networks should perhaps be included in relapse prevention discussions/plans undertaken at the end of the coaching relationship.

Accountability to self and others was an important facilitator of long‐term behavior change both during HWC and afterward. This finding is supported by Nelson et al. (2020) who found that accountability and self‐efficacy are valuable components of HWC. Within our study, participants who made themselves accountable to others and took personal responsibility for their health had greater success in maintaining healthy behaviors. Conversely, a lack of accountability and personal responsibility was associated with disengagement and relapse. Promoting accountability may therefore enhance the effectiveness of HWC for stroke prevention. As many participants in this study were “paying it forward” via leadership roles or informally coaching others, encouraging clients to support behavior change in others may strengthen and reinforce motivation to maintain change themselves.

The authors did not identify any differences in experiences based on CVD risk, gender, ethnicity, or age. The timing of the intervention, self‐awareness, community engagement, and accountability appeared to be more significant factors in the achievement of long‐term behavior change than personal characteristics, such as age, gender, ethnicity, or CVD risk.

## 6 CONCLUSION

HWC may provide a platform for addressing multiple facilitators of long‐term health behavior change. Conversations that explore and enhance the awareness of personal risk and the impact of potential disease, accountability, happiness, life satisfaction, self‐efficacy, emotional well‐being, community engagement, and social supports may be useful in facilitating long‐term behavior change. Delivering interventions at an appropriate time and being responsive to the needs of the person and their contextual demands may strengthen the effectiveness interventions. Furthermore, the use of tools and the development of new skills to support decision‐making and problem‐solving may aid the maintenance of healthy behaviors post intervention. Potential barriers and pitfalls to avoid would be poor timing of interventions, making assumptions about participant preferences on cultural safety or not consulting participants on this, and focusing exclusively on physical health rather than being person‐centered and responsive. There is increasing awareness and use of HWC in primary care as a tool to enhance health and well‐being beyond the medical model of prescribing medications and offering basic lifestyle advice. Hence, the findings of this study extend beyond the current study and may be used to inform the content and delivery of future HWC programs for stroke prevention, specifically around the potential barriers and facilitators of long‐term behavior change.

### 6.1 Strengths and limitations

This study has several strengths, including utilizing an existing cohort of HWC participants at increased risk of CVD and across a range of ages; the use of qualitative semi‐structured interviews to capture real‐life thoughts and experiences of people that have undertaken HWC for stroke prevention and using methodology that allowed for rigorous and reflexive analysis of the data, therefore ensuring that interpretation and conclusions reflected the participant experience. Furthermore, the COREQ checklist (Tong et al., 2007) was utilized in order to further strengthen the methodology and ensure the comprehensive reporting of the study findings. The limitations of this study include the relatively small sample size and the possibility of recall bias given the time between last coaching session and interview. Furthermore, although the sample was fairly ethnically diverse and included a good number of indigenous people, the Chinese Asian population are not well represented in this study, thus limiting the external validity of the findings to people of diverse ethnic and cultural backgrounds. More purposive sampling in the recruitment process may have ensured diversity were reached and therefore strengthened the study and maximized its validity. Despite these limitations, the study has clinical and research utilities as it provides valuable insight into the barriers and facilitators of long‐term behavior change from the perspective of study participants.

## CONFLICT OF INTEREST

The authors have no conflict of interest.

## Data Availability

Anonymized data will be available on request.
